# Exogenous nutrients and carbon resource change the responses of soil organic matter decomposition and nitrogen immobilization to nitrogen deposition

**DOI:** 10.1038/srep23717

**Published:** 2016-03-29

**Authors:** Ping He, Song-Ze Wan, Xiang-Min Fang, Fang-Chao Wang, Fu-Sheng Chen

**Affiliations:** 1 College of Forestry, Jiangxi Agricultural University, Nanchang 330045, China; 2 Collaborative Innovation Center of Jiangxi Typical Trees Cultivation and Utilization, Jiangxi Agricultural University, Nanchang 330045, China

## Abstract

It is unclear whether exogenous nutrients and carbon (C) additions alter substrate immobilization to deposited nitrogen (N) during decomposition. In this study, we used laboratory microcosm experiments and ^15^N isotope tracer techniques with five different treatments including N addition, N+non-N nutrients addition, N+C addition, N+non-N nutrients+C addition and control, to investigate the coupling effects of non-N nutrients, C addition and N deposition on forest floor decomposition in subtropical China. The results indicated that N deposition inhibited soil organic matter and litter decomposition by 66% and 38%, respectively. Soil immobilized ^15^N following N addition was lowest among treatments. Litter ^15^N immobilized following N addition was significantly higher and lower than that of combined treatments during the early and late decomposition stage, respectively. Both soil and litter extractable mineral N were lower in combined treatments than in N addition treatment. Since soil N immobilization and litter N release were respectively enhanced and inhibited with elevated non-N nutrient and C resources, it can be speculated that the N leaching due to N deposition decreases with increasing nutrient and C resources. This study should advance our understanding of how forests responds the elevated N deposition.

Elucidation of the process of decomposition is vital to our understanding of the functioning of forest ecosystems, and to predict the consequences of global environmental changes on carbon (C) budgets^1^,^2^. Increasing concentrations of CO_2_ in the atmosphere are a growing cause for concern. Organic matter decomposition is the most important source of atmospheric CO_2_ from terrestrial ecosystems; thus, it is necessary to gain a better understanding of decomposition processes and the relevant control mechanisms^3^. On the other hand, decomposition is closely allied to nutrient cycling, and is essential for the release of organically-bound nutrients, which provide plant-available nitrogen (N) and other elements requited for plant growth in terrestrial ecosystems^4^. Decomposition is responsible for huge amounts of the CO_2_ returned to the atmosphere and for the formation of humic substances that contribute to soil fertility as well as long-term C storage^5^. Clearly, the process of forest soil and litter decomposition require further investigation, especially since it occurs mainly on or below ground and is largely “out of sight”^2^.

The global increase in N deposition is expected to alter soil organic matter and litter decomposition and ultimately, to affect forest ecosystem C storage and nutrient status^6^,^7^. Nitrogen deposition has been found to increase, decrease or have no effect on decomposition^8^, while soil and litter responses to N deposition often differ^9^. Saiya-Cork^10^ found that N deposition can accelerate litter decomposition and depress soil organic matter decomposition. This indicates that N deposition has an impact on the net soil CO_2_ evolution through its different effects on litter and soil organic matter decomposition, and thus alters forest floor C storage and nutrient availability.

Nitrogen effects on decomposition are influenced by internal N concentration and external nutrient conditions^6^,^9^. It is verified that aspects of litter chemical quality (such as C/N) dominate the decomposition rate within a single climate zone^1^. However, the effects of the exogenous nutrient supply and its interaction with endogenous nutrients on organic matter decomposition are still controversial^4^,^11^. For example, Hobbie^12^ found that the effects of substrate N and exogenous N supply on decomposition were inconsistent and exogenous N supply was not related to litter N concentration. A meta-analysis conducted by Janssens^13^ showed that N deposition in general, impeded soil organic matter decomposition in temperate forest, and suggested that the potential reduction effect in tropical forests required further investigation due to the differences in soil age and properties such as N availability.

In addition to exogenous N supply, other nutrients, especially P, K, Ca and Mg, are also important elements that influence the decomposition process, although few studies have examined the potential regulatory role of these non-N nutrients in decomposition dynamics^4^,^14^. Similar to N, increasing exogenous P supply has been found to have either positive, negative or no effect on decomposition rates^12^. Chen *et al*.^4^ found that P addition decreased the percentage of *Castanopsis sclerophylla* foliar litter C retention, but had no effect on N release during a 540 days field experiment. In contrast, P addition increased *Pinus massoniana* foliar litter N release rate, but had no effect on C retention. Furthermore, the effect of the mixed addition of C, N and P on C and N dynamics differed from the effects of the individual element treatments for litters of both species.

Such conflicting results are generally explained by the mechanism in which heterotrophic microbial activity without growth requires only few nutrient resources to maintain auto-respiration, while active growth requires a C:N:P stoichiometry of the microbial population that is closely matched by the stoichiometry of resource uptake^15^,^16^. Clearly, the effects of nutrient and carbon additions on exogenous N immobilization by litter or soil organic matter requires elucidation^11^ in order to unravel the role of N deposition in shaping the decomposition rate, C and N dynamics^3^ as well as forest N leaching and water pollution.

In addition, it has been hypothesized that the microbial activity responsible for organic matter decomposition is nutrient-limited during the early stages when labile C compounds are abundant, but is limited by low C quality (e.g., high lignin and cellulose) during the late stages^2^. Therefore, it is important to study the effect of C addition on litter and soil organic matter decomposition. The ratio of carbohydrate to nutrients is a good index reflecting the change in organic matter mass, and C consumption and N releases^17^. Such information is key to understanding the effects of exogenous nutrients and C input on decomposition and its responses to atmospheric N deposition^6^. How the addition of nutrients and C alters the N deposition effects on soil organic matter and litter decomposition rate and nutrient dynamics, especially the fate of exogenous N addition with decomposition, remains to be determined. Some research has indicated that organic matter N retention increases with decomposition stage regardless of exogenous N input^18^. However, the source of immobilized N in organic matters is still unclear, although it can be identified using ^15^N Isotope tracer techniques^19^. In general, the effects of non-N nutrient, C source and N deposition and especially their interactions on soil organic matter and litter decomposition processes are complicated, and we need identify the biological (such as microbial immobilization) and abiotic (nutrients supply and their stoichiomoistry) contributions to mediate decomposition.

As an important native fast-growing and commercial tree species, Chinese fir (*Cunninghamia lanceolata*) has been widely planted in subtropical regions and the management practices can be traced back 1,000 year in southern China^20^. With industrialization, more intensive agriculture management and human population growth, atmospheric N deposition has become one of the most important environmental factors controlling ecosystem function and structure in southern China^21^. The impact of N deposition on forest C and nutrient cycling in Chinese fir plantations and the feedback function (forest regulation capacity to N deposition) in subtropical China are poorly understood^3^, and the interactions among exogenous non-N nutrients, C and N deposition on soil organic matter and foliar litter decomposition have not yet been reported. However, these information should help understanding how forests respond to elevating N decomposition.

We designed a one-year soil organic matter and litter decomposition experiment to reveal the coupling effects of non-N nutrient and glucose addition, and N deposition using 99.99% ^15^N isotope tracer. Both CO_2_ emission and immobilized ^15^N were measured and identified, respectively at three different periods including early, middle and late stages. Our major hypotheses are: 1) N deposition can significantly increased the CO_2_ emission and N mineralization of the forest floor (soil and litter) in the subtropics; 2) exogenous non-N nutrient and C input can stimulate the effect of N deposition on soil organic matter and litter decomposition; 3) N immobilization capacities to atmospherically deposited N are different between soil organic matter and foliar litter, and the magnitude is dependent on exogenous non-N nutrient and C resources, and decomposition stage. Our results will also provide information for the development of countermeasures to deal with the potentially negative effects of N deposition on soil carbon stability and nutrient dynamics (such as N leaching) in subtropical forest regions of China.

## Results

### Soil and litter CO_2_ emission rates and litter mass remaining percentage

Nitrogen addition alone [N] and in combination with non-N nutrients [N+nutrients] significantly decreased CO_2_ emission rates in soil (70% and 72% lower than the control, respectively) and litter (35% and 31% lower than the control, respectively) across the whole decomposition period (Table 1). There were no significant differences in the average soil and litter CO_2_ emission rates between [N] and [N+nutrients], while [N] had distinct lower CO_2_ emission in both soil and litter than in [N+carbon] and [N+both] (Table 1). In contrast, CO_2_ emission rates following [N+both] treatment were significantly lower than those following [N+carbon] treatment during both soil and litter decomposition, although no significant difference was observed during the late stage of litter decomposition (Table 1). Meanwhile, litter mass remaining percentage was generally highest under [N+carbon] and [N+both] treatments, followed by [N+nutrient] and [N], and lowest for CK (Table 1).

**Figure 1:**
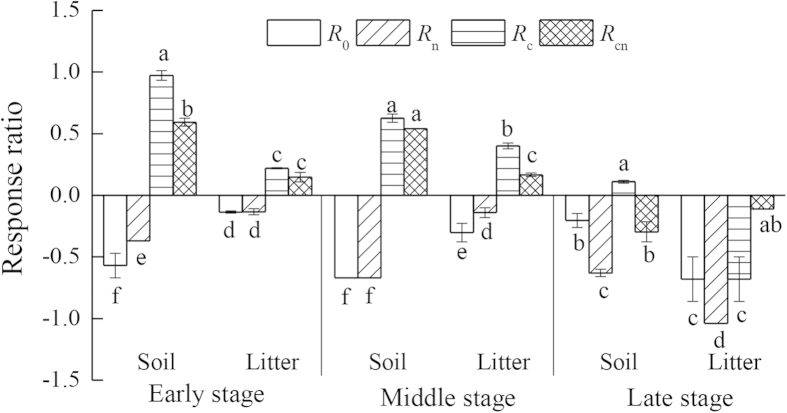
Response ratio of soil and litter CO_2_ emission rates to N addition during the early, middle and late decomposition stages under various treatments. Note: error bar represents one standard error. Different letters indicate significant (*P* < 0.05) differences among four treatments. *R*_n_, *R*_c_, *R*_cn_ and *R*_0_ represent the size of the effects of N deposition following the addition of non-N nutrients, C, both C and non-N nutrients, and without the addition of either C or non-N nutrients.

Similarly, the response ratio of CO_2_ emission to N deposition (*R*) was significantly influenced by substrate type, exogenous input, decomposition stage, and their interactions (Table 2). Both soil and litter *R*_0_ and *R*_n_ were less than 0, while *R*_c_ and *R*_cn_ generally changed from >0 during the early and middle stages to <0 during the late stages (except soil *R*c = 0.11). The *R* variation ranges were generally larger for soil than for litter, and larger following C addition than following nutrient addition. In general, the *R* variation ranges reduced from the early and middle stages to the late stage (Fig. 1).

### ^15^N immobilized by soil and litter

Immobilized ^15^N was generally higher in soil than in litter (Table 3). Compared with [N] treatment, soil ^15^N increased with the addition of other nutrients and C except during the late decomposition stage under [N+nutrient] treatment. Litter ^15^N decreased during the early stage but increased during the late stage with non-N nutrient and C addition, while no significant difference was detected during the middle stage (Table 3).

**Figure 2:**
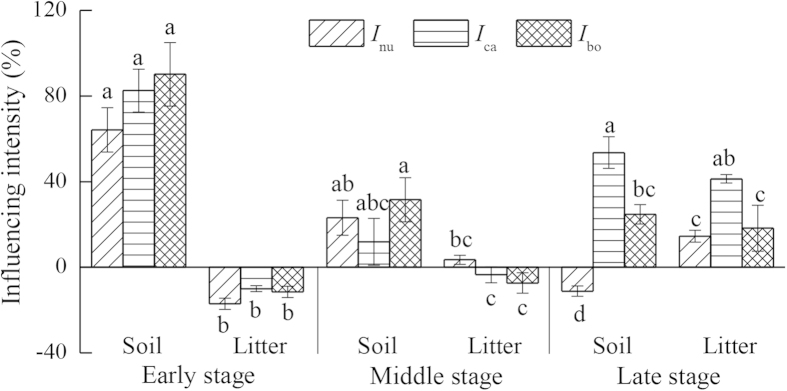
Influence intensity on soil and litter ^15^N immobilization during the early, middle and late decomposition stages under various exogenous input treatments. Note: Mean ± 1 standard error. Different letters indicate the differences among three treatments in soil or litter. *I*_nu_, *I*_ca_ and *I*_bo_ represent the influence intensity of the addition of non-N nutrients, C, and both C and non-N nutrients, respectively.

The ^15^N immobilization (^15^N-*I*) was also significantly influenced by substrate type, exogenous input, decomposition stage, and their interactions (Table 2). During the early and middle stages, ^15^N-*I* was much greater in soil than in litter, with no significant differences among the three treatments with nutrient and C additions. In contrast, there were generally no differences in ^15^N-*I* between soil and litter during the late stage. Moreover, ^15^N-*I* during the late stage was higher under [N+carbon] treatments than that under [N+nutrient] and [N+both] treatments for both soil and litter, and higher in soil under [N+both] than that under [N+nutrient] treatment, while no differences were observed in ^15^N-*I* of litter for both treatments (Fig. 2).

### Mineral N extracted from soil and litter

**Figure 3:**
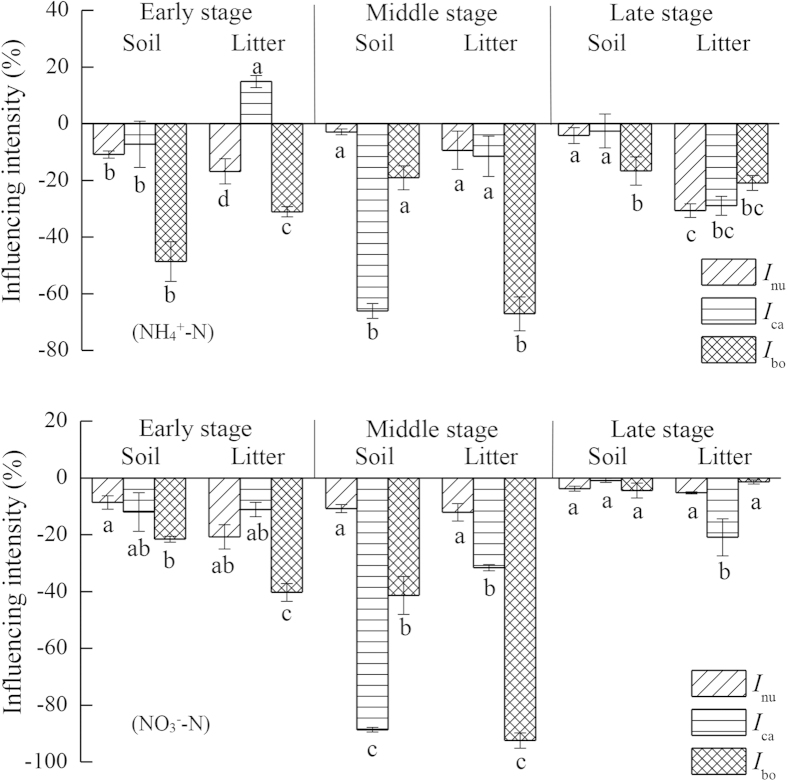
Influence intensity of different exogenous input treatments on extractable mineral N (NH_4_^+^-N and NO_3_^−^-N) in soil and litter during the early, middle and late decomposition stages. Note: Mean ± 1 standard error. Different letters indicate the differences among three treatments in soil or litter. *I*_nu_, *I*_ca_ and *I*_bo_ represent the influence intensity of the addition of non-N nutrients, C, and both C and non-N nutrients, respectively.

Extractable mineral N obtained from soil and litter significantly increased due to exogenous N addition (Table 4). The influences on NH_4_^+^-N and NO_3_^−^-N varied with substrate type (except NH-*I*), exogenous input and decomposition stage, as well as their interactions (Table 2). Both NH-*I* and NO-*I* were less than 0 except for the litter under [N+carbon] treatment during the early stage. The absolute values of NH-*I* and NO-*I* were larger under [N+both] treatment than those under [N+nutrient] and [N+carbon] treatments during the early decomposition stage. In contrast, both NH-*I* and NO-*I* were much higher in soil under the [N+carbon] treatment than those under [N+nutrient] and [N+both] treatments in litter, both NH-*I* and NO-*I* were much higher under [N+both] treatment than those under [N+carbon] and [N+nutrient] treatments at the middle decomposition stage. Additionally, both NH-*I* and NO-*I* showed only a slight difference among the three treatments during the late decomposition stage (Fig. 3).

For soil, the average influence intensity was larger under [N+both] treatment (−39.72 ± 7.27% and −44.73 ± 13.24% for NH-*I* and NO-*I*, respectively) than that under [N+nutrient] treatment (−18.97 ± 3.97% and −12.70 ± 2.71%, respectively) and under [N+carbon] treatment (−8.53 ± 6.79% and −16.92 ± 9.13%, respectively). For litter, the average influence intensity was much smaller under [N+carbon] treatment (−6.01 ± 1.55% and −7.74 ± 1.34%, respectively) than that under [N+nutrient] treatment (−25.31 ± 10.64% and −33.85 ± 13.94%, respectively) and [N+both] treatment (−28.17 ± 5.84% and −22.45 ± 5.74%, respectively) (Fig. 3 and Table 4).

## Discussion

### Negative effect of N deposition on decomposition rate

N deposition has become a recent focus of research, and the influence of N deposition on organic matter and litter decomposition has been widely explored^3^,^7^,^9^,^13^; however, the magnitude and direction of the influences are under debate due to the complexity and invisibility of the decomposition process^2^. In our study, the CO_2_ emission rates of both soil organic matter and litter decreased by 66% and 38%, respectively, in response to N addition, compared with the control. Meanwhile, mass remaining percentage of litter treated by N deposition was 11%, 10% and 18% higher than CK treatment at the early, middle and late decomposition stages, respectively. This inhibitory effect has been observed in many studies^22^,^23^, although N enrichment might have no significant effect on average decomposition rate across a large region^6^,^7^. It is well-known that microbes are the main decomposers of soil organic matter and litter^16^,^24^. Many studies have shown that microbial biomass and activity decrease with the addition of N to soil and litter. For example, Compton *et al*. found that soil microbial biomass and diversity significantly decreased due to N additions at Harvard forest^25^. Frey *et al*. observed that N addition resulted in a decrease in active fungal biomass and the activity of the lignin-degrading enzyme in a temperate hardwood and pine forest^26^. In our study, we also observed less abundant mycelium and mildew in soil and litter in response to N addition compared with that observed without N addition during the early and middle stages (unpublished data, See Fig. S2 for decomposing litter during the early stage). Clearly, all these phenomena supported our speculation about microbial response to N addition.

Our results show that the inhibition intensity with N addition varied with substrate quality and decomposition stage^9^. Generally speaking, N addition tends to reduce the decomposition rate of substrates with lower C/N ratios^4^. It is well known that the initial C:N ratio is higher in the litter (71:1 on average) than that in the microbes (7:1 on average), exogenous N may inhibit synthesis of ligninolytic enzymes^22^,^27^ or react with products of lignin degradation to form other recalcitrant compounds in N-rich forests^28^,^29^. Therefore, it is not surprising that the inhibitory effect of N addition was higher for soil than for litter, especially during the early stage of decomposition.

On the other hand, our results showed differences in the temporal pattern of the N addition effect between soil and litter. The inhibition percentage of soil organic matter decomposition caused by N addition was much higher during the early (71%) and middle stages (78%) than during the later stage (36%), while the inhibition percentage of litter decomposition was lowest during the early stage (27%), followed by the middle stage (49%), and highest during the late stage (74%). We speculate that the rate of soil organic matter decomposition is largely dependent on labile C concentration, which decreases with decomposition time, and N addition could accelerate CO_2_ emission in soil organic matter, mostly from labile C^30^. In contrast, litter decomposition might be regulated mainly by N and lignin concentrations, which increase with decomposition time, and N could reduce the decomposition rate to a greater extent in the later stages, when the concentrations of N and lignin in litter increase, and lignin degradation predominates^31^. Clearly, the effect of N addition on substrate decomposition correlates closely with internal quality factors, such as N concentration and C/N. It can be speculated that the negative interactions between decomposition rate and N addition observed in our study are dominated mainly by labile C concentrations in soil organic matter^30^ and lignin and N concentrations in litter^32^.

### Influence of exogenous nutrient and carbon supply on N deposition effect

In addition to the effects on internal quality, exogenous environmental conditions, especially nutrient availability and carbon sources are also important factors driving the common pattern of substrate decomposition in response to N addition^4^,^23^,^33^. The influences of exogenous nutrient and C source manipulation on soil organic matter and litter decomposition exist almost everywhere on Earth^11^. In this study, we simply used the addition of NH_4_NO_3_ alone and in combination with C_12_H_22_O_11_ and P+K+Ca+Mg to simulate N deposition, and changed exogenous non-N nutrients and the available C source, and their stoichiometry in order to understand the influence of exogenous environmental condition on the effects of N addition.

Our results showed that exogenous non-N nutrients tended to decrease the N deposition effect (absolute difference) on soil organic matter decomposition rate during the early stages, but increased during the late stage, with no differences observed during the middle stage, irrespective of the addition of exogenous available C. Nutrient availability is a key factor in the regulation of soil CO_2_ emission^13^,^23^. Microbial heterotrophs in soil are highly responsive to altered nutrient availability. Nutrient input, in particular, modifies the stoichiometry of soil N and other nutrients, which in turn affects decomposer activity and growth, and the processes of organic matter mineralization^23^,^34^,^35^. In general, the mixture of N and other nutrients improved the stability of soil organic matter during the early stages, although this function decreased with increasing decomposition time, and showed the opposite tendency during the late stage due to the difference in mineralizable C amounts in soil treated by the addition of N ([N] and [N+carbon]) and the mixture of N and non-N nutrients ([N+nutrient] and [N+both]).

In contrast, exogenous non-N nutrients did not alter the effect of N deposition on the litter decomposition rate during the early stage, while the rate was decreased during the middle stage irrespective of exogenous available C addition, and decreased during the late stage only in response to the addition of the mixtures with C. In general, non-N nutrient addition showed a minimal change in the effect of N deposition on the litter decomposition rate, although the coupling effect on increasing stability of exogenous available C requires confirmation^36^. A full-factorial C, N, and P fertilization experiment, showed that the addition of P or C alone did not alter the litter decomposition rate in the subtropical forest ecosystem^3^, although the soil P content was very low, and litter C quality (C/N ratio) controlled the decomposition^4^. However, the combined amendments of nutrient and C (energy supply of decomposers) significantly increased litter mass loss, which indicates that nutrient and C supply co-determined the stimulation of decomposition by nutrient addition. Afterwards, providing decomposer organisms with the extra energy required for the metabolic breakdown decomposition products. Therefore, we deduced that N might control the Chinese fir litter decomposition process, which is supported by the key role of lignin during litter decomposition^31^. Furthermore, the interactive effect of C and P also played an important role, which could be explained by the stoichiometric balance of decomposer requirements^17^. Thus, it is not surprising that litter mass remaining percentage was lower under the nutrient addition treatment than the C addition treatment, since the Chinese fir litter has lower P concentration, and much higher C/P ratio (1284) than soil and most tree litters^17^.

Finally, the increasing magnitude of CO_2_ emission due to exogenous C addition was much higher in soil than in litter, which might be explained by the priming effect of new C input on old organic C decomposition^37^, since soil organic matter exists lots of old OC due to long-term litter input, but all OC in litter are fresh. If this is true, it is not surprising that the orders in the decomposition rates of soil organic matter and litter associated with the different treatments during the late stage were opposite to those observed during the early and middle stages (tradeoff for C loss during different stages).

### Nitrogen immobilization and release by soil organic matter and litter

It is well-known that environmental N can be taken up and conserved by the soil and litter^19^,^38^,^39^. However, the magnitude of the retention following the addition of exogenous N into soil organic matter and litter is seldom reported; this parameter can be determined accurately using ^15^N tracing methods^19^,^40^. Our results showed that soil organic matter could immobilize more atmospherically deposited N than litter. Rapid biological^41^ and abiotic immobilization^42^ could both play important roles in the high soil organic matter retention efficiency of deposited ^15^N, while litter N immobilization by abiotic processes is generally low.

Exogenous N was immobilized in soil organic matter at much higher levels during the middle stage than during the early and late stages, while the levels were much lower in litter during the early stage compared with those in the middle and late stages. Several studies have shown that N tends to be associated with substrate lignin^29^,^31^,^32^. A ^15^N-labeled beech litter experiment showed that almost all external N, including N release from litter, is incorporated into the decomposing litter during the first year^40^. Berg also revealed that the concentration of N in the lignin fraction increased linearly with the loss of mass as decomposition proceeded^43^,^44^. The results reported by Axelsson and Berg^45^ also suggested that, in more N-rich substrates, the binding of N to more recalcitrant forms may take place at a higher rate. This explains the observation made in our study showing that the immobilized ^15^N increased from the early stage to the middle stage due to the cumulative effects of ^15^N addition at 0, 45 and 90 days. Interestingly, the immobilized ^15^N decreased rapidly in soil organic matter, but remained fairly constant in litter from the middle stage to the late stage, when N addition had stopped after 90 days. Therefore, we thus speculate that the dynamics of litter lignin activity might be an important mechanism by which N is preserved in a forest ecosystem^29^.

Understanding the retention of N input by forest soil and litter is a critical issue in the context of global biogeochemical and climate changes^39^,^40^; therefore, the ability to manipulate this process by human management approaches, such as fertilization, requires investigation^46^. Nitrogen immobilization into decomposing soil organic matter and litter would be enhanced by both nutrient supply and demand for C source by decomposers^5^. Our results show that the addition of nutrients or carbon generally increased soil retention of exogenous N, while the effects on litter ^15^N immobilization varied with decomposition stage, being negative, neutral and positive, respectively, during the early, middle and late stages (a positive average effect across the 1 year incubation period). The differences in ^15^N in the decomposing soil organic matter and litter would be mainly due to rapid abiotic immobilization in soil^42^ in addition to biological immobilization in both soil and litter^41^. These results are highly consistent with the observation that soil extractable mineral N decreased with the addition of nutrients and carbon, but are contradictory to the observation of decreasing litter extractable mineral N with the addition of nutrients and carbon, especially during the early stages. Thus, we speculate that internal N release from litter could be inhibited by the mixed addition of non-N nutrients and carbon^40^,^45^.

Our results also showed that the intensity of the negative influence of the addition of non-N nutrients and carbon on extractable mineral N was in general stronger in soil treated by [N+both] than by [N+nutrient] and [N+carbon], but was much higher in litter treated by [N+nutrient] and [N+both] than by [N+carbon]. These results indicate that stimulation of soil microbial N immobilization by C amendment (energy supply) is co-determined by non-N nutrient (P+K+Ca+Mg) fertilization, which might have a better C:N:P stoichiometry in available forms^47^. On the other hand, litter internal N release was inhibited, while the N immobilization increased following exogenous C addition, which satisfies the energy of decomposers^3^,^11^, although the negative effect of simulated N deposition dominated the litter N dynamics in our study. Chen *et al*.^4^ found that exogenous C addition improved litter N concentrations and N retention in organic matter, and indicated that carbon quality (exogenous C_12_H_22_O_11_ and litter C fractions) regulates N dynamics in decomposing litter^36^, although the magnitude also correlated closely with the availability of N and other nutrients in microhabitats, such as soil vs. litter. Taken together, our results indicate that the potential N leaching in these forests due to N deposition decreases with increasing nutrient and C resources, since exogenous N immobilized by soil was enhanced, and litter internal N release was reduced with elevated nutrient and C resources.

Due to the warm, humid climate, subtropical forests are characterized by high rates of soil organic matter and litter decomposition, which dominate the C and N balance in these ecosystems^11^,^33^. On the one hand, N retention capacity is enhanced by the decomposing litter, as decomposition provides an abundant C source and thus, stimulates microbial N demand, resulting in a decrease in N availability and potential N losses^18^,^19^. On the other hand, soil organic matter increases the efficiency of deposited N on biological immobilization^41^ and abiotic immobilization^42^, when the forest reaches the situation of elevated N deposition level. That is to say, the elevated N immobilization due to the decomposition of soil organic matter and litter reduces atmospheric N deposition that accumulates in the forest floor. However, it should be noted that this effect of enhanced N immobilization might be uncertain in field situation, because organic substrate decomposition-induced N immobilization can be influenced by plant growth^39^.

## Conclusions

This is the first experimental investigation of the mechanism by which non-N nutrient and carbon resources alter the effect of N deposition on soil organic matter and litter decomposition in subtropical Chinese fir forest plantation. We found that N deposition decreased the decomposition rate in both soil organic matter and litter, and the combined effect of N deposition and non-N nutrient addition on CO_2_ emission rate differed with substrate type, carbon input and decomposition period. Exogenous C, non-N nutrient resources and their interaction would alter the effects of N deposition on forest floor C and N dynamics in subtropics. The risk of N leaching in the forest due to N deposition decreases with increasing C and nutrient resources, since exogenous N immobilized by soil was enhanced and litter internal N release was reduced with increasing C and nutrient resources. Meanwhile, litter decomposition rate was decreased by N deposition with increasing C and nutrient, but the extent may be highly context-dependence, since litter CO_2_ emission significantly higher in [N+both] than in [N], while [N] distinctly decreased soil and litter CO_2_ emission. Based on our results, we suggest that residual remaining and non-N fertilization would be effect practices to improve atmosphere N and other elements utilization in subtropical Chinese fir plantation.

## Methods

### Experimental materials

We collected soil (0–15 cm) and litter samples in a 15-year old Chinese fir plantation at Qianyanzhou Ecological Research Station, Chinese Academy of Sciences (26°43′ N, 115°04′ E) in March 2013. The region has a subtropical moist monsoon climate, with a warm, dry summer and a cool, wet winter. The soil is Typic Hapludult Ultisols (locally “red soil”), which develops from Quaternary Red Clay and covers over 60% of 1.14 million km^2^ of total land area in the southern China^48^. The average stand density, diameter at breast height and height of the selected Chinese fir plantation are approximately 1950 tree/ha, 10 cm and 9 m, respectively.

Topsoil (0–15 cm) was collected at 30 randomly selected points using 2.5 cm soil cores, and mixed as a sample within a 50 × 60 m plot. The roots and organic debris were removed from the soil samples, air-dried, and stored for incubation as well as analysis of organic C, total N and total P^49^. Foliar litter was obtained using 1 × 1 m litterfall boxes under 30 tree canopies in the selected plot from December 2012 to March 2013. All foliar litters were mixed, over-dried at 60 °C and stored for nutrient analysis and incubation. The general properties of soil and litter are listed in Table 5.

### Experimental design

A laboratory microcosm experiment was designed to study the interactive effect of non-N nutrient (P, K, Ca and Mg so on) and C (glucose) additions, and N deposition on the C and N dynamics of soil organic matter and litter decomposition. Soil and litter samples (10 g) were incubated in two 500 ml PVC jars at room temperature (see Fig. S1) from April 2013–April 2014. Five different treatments were applied: N addition alone (60 mg ^15^N per jar; [N]), N and non-N nutrient additions (60 mg ^15^N + 6 mg P + 50 mg K + 30 mg Ca + 10 mg Mg per jar; [N+nutrient]), N and carbon addition (60 mg ^15^N + 500 mg C per jar; [N+carbon]), N + non-N nutrients+carbon ([N+both]), and control (No addition; CK).

In our study, a solution of ^15^NH_4_^15^NO_3_ (99.99% ^15^N abundance) was used as the N deposition treatment to measure soil and litter N immobilization. Non-N nutrients comprised KH_2_PO_4_, MgSO_4_ and CaCl_2_ solutions. Glucose solution was used as the C addition. These solutions were added to the corresponding incubation jars at days 0, 45 and 90 of the experiments. Soil moisture content was adjusted to approximately 40% of the soil water holding capacity by adding distilled water; 10 ml distilled water was added in the litter jar initially and maintained at a constant weight by adding distilled water every 3 days. Nine replicates were used for each treatment (90 jars in total).

Briefly, the first objective of our experimental design is to test the potential effect of N on decomposition rate by comparing CK and [N], and the second is to test N immobilization influenced by non-N nutrient and C addition using a 2 × 2 factorial design.

### CO_2_ emission measurement

CO_2_ emission was measured by closed-jar alkali-absorption to assess soil mineralization and litter decomposition. Small vials containing 5 ml of 1 M NaOH solution were placed in the jars to trap the CO_2_ gas produced during days 88–90 (representing early stage), days 177–180 (middle stage) and days 356–360 (late stage). The quantity of CO_2_ produced was measured by titrating NaOH to pH 8.3 with 0.5 M HCl in the presence of BaCl_2_. The net amount of CO_2_ produced from the soil or litter was calculated as the difference between the treatments and control jars^48^,^50^. Three replicates were applied for each treatment per incubation temperature.

### Exogenous N immobilization and extractable mineral N

After CO_2_ emission measurement, the soil and litter samples (three replicates per treatment) were also used to analyze extractable mineral N and ^15^N immobilization. Samples were mixed with 100 ml 2 M KCl, shaken for 15 min, and immediately separated into the supernatant and solid substrate (soil or litter) by filtration. NH_4_^+^-N and NO_3_^−^-N in the supernatant were measured using the automated ion analyzer (EasyChem Plus, Italy). Soil and litter were over-dried to pass through 0.5 mm sieve before ^15^N measurement. The ^15^N abundance in soil or litter (solid substrate, defined as the immobilized N) was measured using Stable Isotope Ratio Mass Spectrometer (Vario EL III/Isoprime, Germany).

### Nitrogen deposition effect on CO_2_ release

An effect size of N deposition on litter or soil CO_2_ emission was calculated as the natural log of the response ratio *R* = X_n_/X_0_, where X_n_ and X_0_ are the average CO_2_ emission rates with and without N addition, respectively. In our study, *R*_n_, *R*_c_, *R*_cn_ and *R*_0_ represent the size of the effect on N deposition following the addition of non-N nutrients, or C, or both C and non-N nutrients, and without the addition of either C or non-N nutrients.

### Effects of the addition of nutrients and C on exogenous N immobilization and extractable mineral N

The intensity of the influences of nutrient and C addition on exogenous N immobilization and extractable mineral N were calculated as increasing nitrogen percentage *I* (%) = (Y_1_ − Y_0_)/Y_0_ × 100, where Y_1_ and Y_0_ are the average ^15^N abundance and mineral N content, respectively, following the addition of N and non-N nutrients, and C and N alone. In our study, *I*_nu_, *I*_ca_ and *I*_bo_ represent the intensity of the influence of the addition of non-N nutrients, or carbon addition or both carbon and non-N nutrients.

### Statistical analysis

Multi-way analysis of variance (ANOVA) was used to determine the interactions among substrate types (soil vs. litter), exogenous conditions (different treatments with N, non-N nutrient and carbon addition) and decomposition stages (early, middle and late stages). One-way ANOVA and least significant difference (LSD) methods were used to compare the differences among exogenous conditions and decomposition stages. All analyses were performed in SPSS 16.0 for windows (SPSS Inc., Chicago, US). The standard *P* = 0.05 level was used throughout as a cutoff for statistical significance.

## Additional Information

**How to cite this article**: He, P. *et al*. Exogenous nutrients and carbon resource change the responses of soil organic matter decomposition and nitrogen immobilization to nitrogen deposition. *Sci. Rep*. **6**, 23717; doi: 10.1038/srep23717 (2016).

## Supplementary Material

Supplementary Information

## Figures and Tables

**Table 1:** The dynamics of CO_2_ emission (mg CO_2_ g^−1^ OC d^−1^) and mass remaining pecentage (%) from litter or soil treated with nitrogen, nutrients and carbon addition.

Treatments	Early stage	Middle stage	Late stage
Soil CO_2_ emission			
CK	1.04 ± 0.08c	2.08 ± 0.15c	0.50 ± 0.01a
[N]	0.30 ± 0.08e	0.45 ± 0.00d	0.32 ± 0.05ab
[N+nutrients]	0.45 ± 0.00d	0.45 ± 0.00d	0.11 ± 0.01b
[N+carbon]	9.89 ± 0.86a	8.92 ± 0.68a	0.64 ± 0.02a
[N+both]	4.09 ± 0.32b	7.14 ± 0.00b	0.26 ± 0.05ab
Litter CO_2_ emission			
CK	3.02 ± 0.14C	1.00 ± 0.05C	0.31 ± 0.00A
[N]	2.20 ± 0.06D	0.51 ± 0.08D	0.08 ± 0.02AB
[N+nutrients]	2.22 ± 0.13D	0.73 ± 0.07D	0.03 ± 0.00B
[N+carbon]	4.99 ± 0.08A	2.52 ± 0.15A	0.08 ± 0.02AB
[N+both]	4.27 ± 0.38B	1.47 ± 0.04B	0.24 ± 0.00A
Litter mass remaining pecentage			
CK	82.53 ± 0.55D	75.62 ± 0.25C	65.36 ± 4.01D
[N]	91.96 ± 0.46B	83.29 ± 0.59B	77.21 ± 1.66C
[N+nutrients]	85.62 ± 0.29C	80.94 ± 0.42B	81.01 ± 1.90BC
[N+carbon]	94.31 ± 0.29A	91.20 ± 1.53A	90.82 ± 1.31A
[N+both]	92.15 ± 0.70B	91.70 ± 0.07A	86.26 ± 2.57AB

Note: Mean ± 1 standard error. Different lower or upper case letters indicate the differences among five treatments of soil and litter, respectively. CK: Control, [N]: N addition alone, [N+nutrient]: N and non-N nutrient addition (N+P+K+Ca+Mg), [N+carbon]: N and carbon addition, [N+both]: N+ non-N nutrients+ carbon addition.

**Table 2:** ANOVA of the effects of substrate type, exogenous conditions and decomposition stage on response ratio of CO_2_ emission to N deposition, and influence intensity on ^15^N immobilization and extractable mineral N obtained from substrates.

Influencing factors	*F* value			
	CO_2_ emission	^15^N immobilization	Extractable NH_4_^+^-N	Extractable NO_3_^−^-N
Substrate type (ST)	29.84***	119.04***	1.36^*ns*^	9.38**
Exogenous condition (EC)	230.85***	7.79**	34.97***	77.56***
Decomposition stage (DS)	154.70***	14.91***	13.89***	220.46***
ST × EC	40.50***	4.00*	19.17***	40.17***
ST × DS	24.10***	64.36***	15.95***	4.57*
EC × DS	19.07***	7.45***	23.13***	52.75***
ST × EC × DS	20.42***	1.10^*ns*^	25.06***	49.75***

Note: ^*ns*^not significant, *P < 0.05, **P < 0.01, ***P < 0.001.

**Table 3:** Exogenous N immobilization (^15^N, ‰) in litter and soil under different treatments.

Treatments	Early stage	Middle stage	Late stage
Soil			
[N]	21.37 ± 0.86b	74.77 ± 9.11b	31.33 ± 2.60b
[N+nutrients]	34.90 ± 2.23a	92.00 ± 6.12ab	27.83 ± 0.78b
[N+carbon]	38.80 ± 2.14a	83.63 ± 8.17ab	48.07 ± 2.33a
[N+both]	40.43 ± 3.16a	98.37 ± 7.72a	39.07 ± 1.43a
Litter			
[N]	23.00 ± 0.85A	36.23 ± 0.68A	26.60 ± 2.45C
[N+nutrients]	19.17 ± 0.61B	37.47 ± 0.78A	30.47 ± 0.74B
[N+carbon]	20.70 ± 0.31B	34.97 ± 1.44A	37.50 ± 0.51A
[N+both]	20.47 ± 0.62B	33.57 ± 1.69A	31.47 ± 2.84B

Note: Mean ± 1 standard error. Different lower or upper case letters indicate the difference among four treatments in soil and litter, respectively. [N]: N addition alone, [N+nutrient]: N+non-N nutrient addition (N+P+K+Ca+Mg), [N+carbon]: N and carbon addition, [N+both]: N+ non-N nutrients +carbon addition.

**Table 4:** Extractable mineral N (mg N/kg substrate) from soil and litter under different treatments.

Treatments	Early stage	Middle stage	Late stage
Soil			
CK	0.30 ± 0.04c	0.38 ± 0.03d	2.04 ± 0.82b
[N]	13.31 ± 0.06a	12.77 ± 0.24a	10.34 ± 0.09a
[N+nutrients]	12.05 ± 0.15ab	11.53 ± 0.17a	9.92 ± 0.19a
[N+carbon]	11.94 ± 0.95ab	1.87 ± 0.14c	10.12 ± 0.45a
[N+both]	9.15 ± 0.42b	7.90 ± 0.81b	8.98 ± 0.44a
Litter			
CK	0.35 ± 0.08C	0.04 ± 0.01C	0.35 ± 0.14C
[N]	10.70 ± 0.25A	12.25 ± 0.33A	9.08 ± 0.26A
[N+nutrients]	8.57 ± 0.35AB	10.81 ± 0.43AB	7.12 ± 0.15AB
[N+carbon]	10.09 ± 0.61A	8.68 ± 0.15B	6.71 ± 0.41B
[N+both]	6.59 ± 0.23B	1.31 ± 0.20C	7.81 ± 0.14AB

Note: Mean ± 1 standard error. Different lower and upper case letters indicate the difference among five treatments in soil and litter, respectively. CK: Control, [N]: N addition alone, [N+nutrient]: N+non-N nutrient addition (N+P+K+Ca+Mg), [N+carbon]: N and carbon addition, [N+both]: N+non-N nutrients+carbon addition.

**Table 5:** General characteristics of soil and litter in a Chinese fir forest plantation in subtropical China.

Substrate type	Organic C (g/kg)	Total N (g/kg)	Total P (g/kg)	C/N	N/P
Soil	82.20	3.95	0.60	21	6.6
Litter	501	7.37	0.39	68	18.7
