# Supplementary material for: Exogenous nutrients and carbon resource change the responses of soil organic matter decomposition and nitrogen immobilization to nitrogen deposition
Source: Sci Rep. 2016 Mar 29;6:23717. doi: 10.1038/srep23717 (PMC4810377; doi:10.1038/srep23717)

Exogenous nutrients and carbon resource change the responses of soil organic matter decomposition and nitrogen immobilization to nitrogen deposition

Ping He[1, #], Song-Ze Wan[1,2, #], Xiang-Min Fang[1,2], Fang-Chao Wang[1], Fu-Sheng Chen[1, 2, *]

Fig. S1 Daily variations in room temperature (°C) and air humidity (%) during the incubation period from April 15, 2013 to April 10, 2014

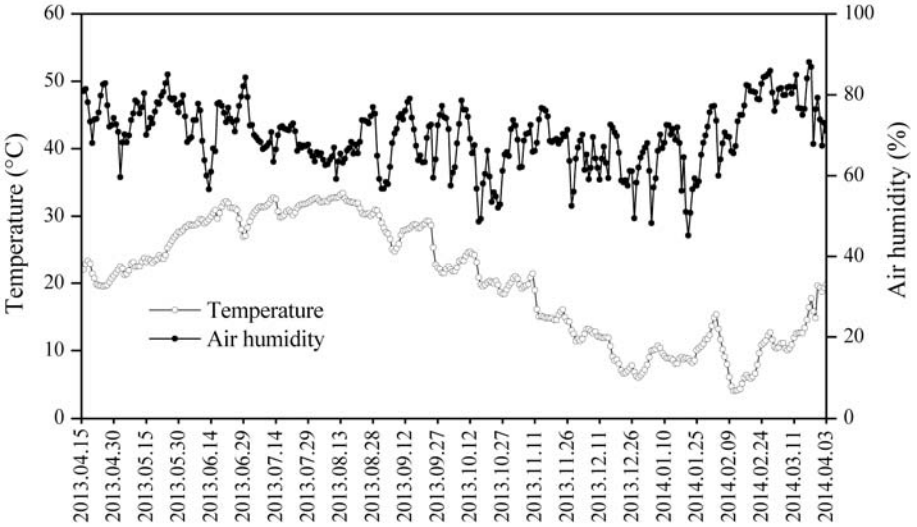

Fig. S2 Mycelium and mildew in decomposing litter during the early stage

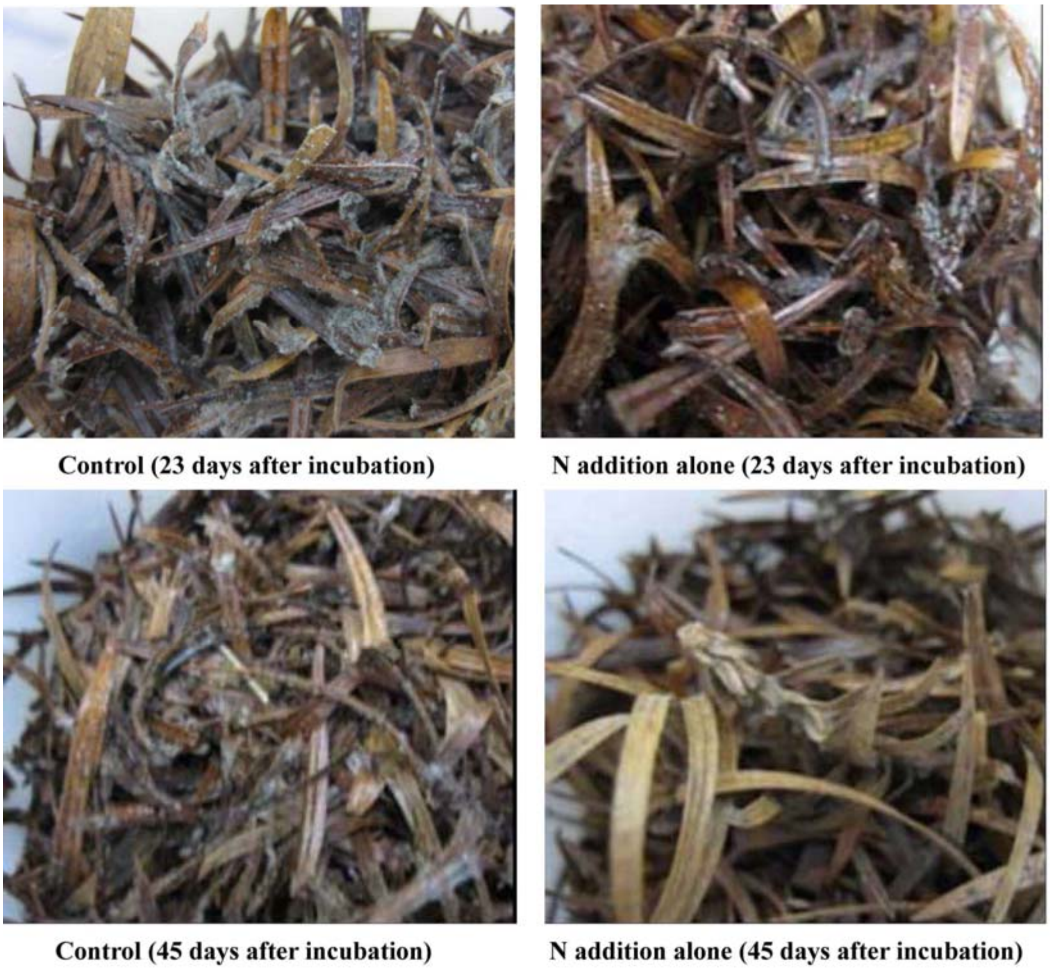

Supplement: Supplementary Information [file srep23717-s1.pdf]
